# DOES CRIMINAL VICTIMIZATION RAISE THE RISK OF IADL LIMITATIONS AMONG OLDER ADULTS?

**DOI:** 10.1093/geroni/igad104.3289

**Published:** 2023-12-21

**Authors:** Jinnan Ren, Madison Sauerteig-Rolston, Mallory Bell, Kenneth Ferraro

**Affiliations:** Purdue University, West Lafayette, Indiana, United States; Purdue University, West Lafayette, Indiana, United States; Purdue University, West Lafayette, Indiana, United States; Purdue University, West Lafayette, Indiana, United States

## Abstract

Many studies have analyzed the effect of victimization on children and young adults, but relatively few have explored its impact on older adults’ well-being. Drawing from cumulative inequality theory, this study analyzed a sample of 11,860 respondents aged 50 and older from the Health and Retirement Study (HRS) to examine whether victimization experiences, measured by physical attack/assault, robbery/home burglary, and fraud, raise the risk of limitations in performing instrumental activities of daily living (IADLs). The analysis, which used zero-inflated negative binomial models to examine the onset and severity of IADL limitations, revealed that criminal victimization was associated with a higher likelihood of IADL limitations (IRR=0.735, p< 0.01), even after adjusting for demographic and health-related factors. Additional analyses examining the influence of each type of victimization showed that the experiences of a physical attack and robbery were related to IADL limitations. These findings demonstrate that criminal victimization experiences are associated with compromised IADL function, especially when the encounter involves an intentional act of immediate harm. There is a need for (a) additional research on the consequences of criminal victimization on the health and well-being of older adults and (b) interventions to help older adults who experience a personal or property crime.

